# Complete Sequence of a *Staphylococcus aureus* Clonal Complex 81 Strain, the Dominant Lineage in Food Poisoning Outbreaks in Japan

**DOI:** 10.1128/genomeA.00853-17

**Published:** 2017-08-24

**Authors:** Yusuke Sato’o, Junzo Hisatsune, Hideki Hirakawa, Hisaya K. Ono, Katsuhiko Omoe, Motoyuki Sugai

**Affiliations:** a Department of Bacteriology, Hiroshima University Graduate School of Biomedical and Health Sciences, Hiroshima City, Hiroshima, Japan; b Kazusa DNA Research Institute, Kisarazu, Chiba, Japan; c Department of Veterinary Medicine, Faculty of Agriculture, Iwate University, Morioka, Iwate, Japan

## Abstract

*Staphylococcus aureus* No. 10 is an isolate from a staphylococcal food poisoning outbreak in Japan, classified as clonal complex 81 subtype 1. It preferentially produces larger quantities of staphylococcal enterotoxin A (SEA) and staphylococcal enterotoxin H (SEH) in foods and media. Here, we report the complete annotated genome sequence of the chromosome and a plasmid.

## GENOME ANNOUNCEMENT

Staphylococcal food poisoning (SFP) is one of the common bacterial food poisonings contracted through the consumption of foods contaminated with toxic amounts of staphylococcal enterotoxins (SEs) produced by *Staphylococcus aureus* (1). We previously characterized isolates from SFP outbreaks in Japan and demonstrated that the clonal complex 81 (CC81) subtype 1 lineage is the major SFP-associated lineage (2). In addition, other researchers reported that a similar lineage was frequently isolated in South Korea (2, 3). These results suggest that this bacterial group is widespread and frequently causes SFP outbreaks in far-east Asia. The major SE genotype of CC81 subtype 1 is *sea seb seh sek seq*. This lineage possesses *sea*-harboring ϕSa3mw2, which is a high-SEA-production-type prophage (4) and produces a large quantity of SEA in media and foods. Strain No. 10 is an isolate from an SFP outbreak in the 1990s in Tokyo, Japan (5), and belongs to CC81 subtype 1 (2). Genomic DNA was extracted with the QIAamp DNA purification kit (Qiagen GmbH, Hilden, Germany). A DNA library was prepared using the Nextera XT DNA library prep kit (Illumina, San Diego, CA). The 250-bp paired-end sequencing of No. 10 was carried out with the MiSeq platform (Illumina). The reads were mapped to the reference genome of *S. aureus* MW2 (GenBank accession no. BA000033) (6) by CLC Genomics Workbench (CLC bio, Arthus, Denmark), with an average 78-read mapping coverage. After the reads were assembled, contigs were aligned to MW2 with OSLay (7). Gap closing was performed by Sanger sequencing. Only the gap within a cell wall protein corresponding to MW2416 remained, because this gene had a long G5-E repeat (8), but we confirmed by PCR that this repeat number in No. 10 was the same as that in strain MW2.

Strain No. 10 had a 2,764,435-bp chromosome containing 2,632 coding sequences (CDSs), 5 rRNA clusters, and 59 tRNAs and six staphylococcal enterotoxins/staphylococcal enterotoxin-like toxins (staphylococcal enterotoxin A [SEA], SEB, SEH, SEK, SEQ, and staphylococcal enterotoxin-like X [SElX]) genes. Compared with the closely related strain MW2 (CC1, a single-locus variant of CC81), No. 10 had an additional *Staphylococcus* pathogenicity island (SaPI [SaPIno10]) harboring *seb* and a putative truncated prophage (ϕSa2no10). Three SE genes, *sea*, *sek*, and *seq*, existed on a prophage similar to ϕSa3mw2. The *seh* and *selx* genes were located on a putative truncated transposon and chromosome, respectively. These locations in No. 10 were the same as those in MW2. Strain No. 10 lacked a staphylococcal cassette chromosome *mec* element (SCC*mec*), Panton-Valentine leukocidin (PVL)-associated prophage (ϕSa2mw2), and two enterotoxin (SEC and SEL)-associated SaPImw2, compared with MW2. Strain No. 10 possessed one plasmid, pNo10, whose length was 20,653 bp. This was classified as a type II plasmid with high similarity to the plasmids of MW2 and MSSA476, both of which were classified as CC1 (6, 9).

Consequently, No. 10 had a genetic background similar to that of CC1, but there were some deletions, insertions, and mutations in some genes, such as enterotoxins, the toxin-antitoxin system, and serine proteases. These differences may contribute to the pathogenesis causing SFP.

### Accession number(s).

The whole-genome nucleotide sequence data for *S. aureus* strain No. 10 have been deposited in DDBJ/EMBL/GenBank under the accession numbers AP015012 and AP015013 for the chromosome and the plasmid (pNo10), respectively.
